# Tashkeela: Novel corpus of Arabic vocalized texts, data for auto-diacritization systems

**DOI:** 10.1016/j.dib.2017.01.011

**Published:** 2017-02-03

**Authors:** Taha Zerrouki, Amar Balla

**Affiliations:** The National Computer Science Engineering School (ESI), Algiers, Algeria

**Keywords:** Natural language processing, Corpus, Arabic language, Diacritization

## Abstract

Arabic diacritics are often missed in Arabic scripts. This feature is a handicap for new learner to read َArabic, text to speech conversion systems, reading and semantic analysis of Arabic texts.

The automatic diacritization systems are the best solution to handle this issue. But such automation needs resources as diactritized texts to train and evaluate such systems.

In this paper, we describe our corpus of Arabic diacritized texts. This corpus is called Tashkeela. It can be used as a linguistic resource tool for natural language processing such as automatic diacritics systems, dis-ambiguity mechanism, features and data extraction.

The corpus is freely available, it contains 75 million of fully vocalized words mainly 97 books from classical and modern Arabic language.

The corpus is collected from manually vocalized texts using web crawling process.

**Specifications Table**

**Table:** 

Subject Area	Computer Science
More specific subject area	Computational linguistics, natural language processing, text to speech, corpus. Arabic language.
Type of data	Text files
How data was acquired	The data is collected from freely published texts in ancients books, these books had been rewritten and vocalized by volunteers manually, to ensure that words are vocalized.
Data format	Raw
Experimental factors	Texts are collected, filtered and converted to text format.
	Texts are cleaned by removing extra spaces and unnecessary stuff. Adding specific description to each book and data.
Experimental features	conduct statistical analysis about word frequencies, for vocalized, semi vocalized and un-vocalized words.
Data source location	N/A
Data accessibility	Data presented in this article is freely available at http://tashkeela.sourceforge.net

**Value of the data**

• This data is very helpful for the statistical training of machine learning algorithms based on natural language processing [2]; It is used by diacritization systems [1], [2], [3], [4], and disambiguation algorithms [4], [5], [6]. It was used in training and in evaluation data as well, and it can be used for similar systems.
• It is used as a linguistic resource to extract features and linguistic data processes, i.e. building lexicons [7], [8], [9], Extraction of Arabic Modal Multiword Expressions [7].
• Furthermore, this data is integrated in many other analysis, like Morphological analysis [10], syntactical models [11], and text-to-speech rule-based extraction [12].
• Extracted texts can be used as samples in learning Arabic language for both beginners and foreigners as in Al-jazeera Learning service [13].

## 1 Data

Data is a collection of Arabic vocalized texts, which covers modern and classical Arabic language. The Data contains over 75 million of fully vocalized words obtained from 97 books, structured in text files.

The corpus is collected mostly from Islamic classical books [14], and using semi-automatic web crawling process.

The Modern Standard Arabic texts crawled from the Internet represent 1.15% of the corpus, about 867,913 words, while the most part is collected from Shamela Library, which represent 98.85%, with 74,762,008 words contained in 97 books (cf. Table 1).

## 2 Experimental design, materials and methods

The process of text vocalization is a hard task to accomplish, however, there are limited vocalized texts, mainly, in learning Arabic language for beginners, or in specific-domains texts like religious texts i.e. Quranic and Hadith scripts. For these reasons, obtaining vocalized texts is considered as very hard task to accomplish [15], [16].

The only resources available to obtain vocalized texts are those religious texts [17], which are often written in classical Arabic, or as new textual scripts written by modern authors who usually use a classical language in general. The classical Arabic language is a bit different from modern standard Arabic, in terms of grammars, vocabularies and semantic [18].

This linguistic feature (language differences) can lead to obsolete evaluation and training of diacritization systems, because most of these systems are supposed to be trained on classical texts, and to be implemented in modern standard Arabic texts.

However, below is a list of available vocalized resource:

• Shamila library^1^:  is an Islamic electronic library which contains hundreds of books in many domains like Hadith ( prophet citation) Fiqh (scientific dogms books), history, preaching, Islamic laws, Arabic language.
  It is freely available in many formats, like websites, desktop applications. these books are rewritten by volunteers and uploaded in suitable format to Shamila library.
  In our case, we count around 97 fully vocalized books, which represent around 75 million words, that form up the main part of Tashkeela corpus data.
• Aljazeera, learning Arabic service
  Aljazeera Network launched a new service to learn Arabic as a foreign language. Aljazeera learning Arabic site [13] provides texts, samples, exercises, courses about Arabic language with many short stories extracted from news. The texts are vocalized to ease reading and facilitate learning process.
  Because it is so difficult to vocalize texts, Aljazeera learning activated the manual review of their automatic diacritization system to ensure a high quality of the generated vocalized texts.
• Maqola, a citation collection: It seeks the best-citation collection in the field of Arabic and Islamic heritage for both past and present, and they display it in fully diacritical format [20].
• Diverse texts crawled from the net

There are a few and limited vocalized texts available online. The reason why, the collection of such texts is also very hard, on the other hand, most of the search engines ignore diacritics in searching process, hence, this disallow users to find vocalized texts online.

To overcome this issue, we managed to use Google verbatim search to find diacritized texts, we have used Google to find diacritics texts without significant keywords to retrieve general texts without any specific keywords, we used most frequent diacritized words [19] which are considered as stop words i.e., . However, we used vocalized stop words as they are not ignored in verbatim search, in case if the writer vocalize them, most probable that the other words in text are vocalized.

The extraction process:

– The Shamela library is basically an e-book reader software, which reads a collection of thousands of books prepared by volunteers. We use Shamela as source to extract vocalized texts.
– We search for vocalized texts in books, by looking up vocalized tags in the book index or keywords i.e.,  (/Fi:/,in),  (/Ila/, to).
– After that we convert crawled texts to certain encoded file format.
– We extract words from text files, in order to count word number and their frequencies (cf. Table 1).
– We then truncate the last short vowel (/Haraka/) from the word, to obtain words without syntactic marks. In most case, the last mark represents the syntactic case like  (/kitab-u/ - a book in subjective case),  (/kitab-a/ - a book in objective case). In other cases, the syntactic mark in not in the end, like  (/kitab-u-ha/, her book).
  We truncate the syntactic mark, in order to count the number of semi-vocalized words and their frequencies ( cf. Table 2).
– We eventually truncate all vowels (Harakat) to count the number of un-vocalized words and their frequencies (cf. Table 2).

## Figures and Tables

**Table 1:** Corpus parts.

Total words	75,629,921	Percent
**Classical Arabic:**	74,762,008	98.85%
– 97 Books filtered from 7079 books from Shamela Library.		
**Modern Standard Arabic**	867,913	1.15%
• 20 modern books	398,911	
• Texts crawled from Internet ○ learning.aljazeera.net ○ al-kalema.org ○ enfal.de ○ diverse …	461,283	
• Manually diacritized	7701	

**Table 2:** Corpus words statistics.

**Feature**	**Count**
Total Word counted:	75 629 921
Arabic vocalized word counted	67 287 202
Punctuation and non Arabic word counted:	8 342 719
Unrepeated un-vocalized word counted:	486 524
Unrepeated vocalized word counted:	998 538
Unrepeated semi-vocalized word counted:	770 702
Estimated number of vocalizations for a word	2.05

## Footnotes

1 http://shamela.ws/
